# Lithotripsy-induced splenic injury requiring emergent splenectomy at a level 1 trauma center

**DOI:** 10.1016/j.tcr.2025.101286

**Published:** 2025-11-17

**Authors:** Matthew B. Goss, Joseph S. Banton, Martha M.O. McGilvray, Stephanie M. Armocida, Arvind Kumar, Douglas J.E. Schuerer, Matthew J. McHale

**Affiliations:** Washington University in St. Louis Mary Culver Department of Surgery, Division of Acute and Critical Care Surgery, 4590 Nash Way (Formerly Children's Place), St. Louis, MO, 63110, United States of America

**Keywords:** Lithotripsy, Splenectomy

## Abstract

**Background:**

An exceptional occurrence, clinically-significant splenic injury secondary to extracorporeal shock wave lithotripsy (ESWL) is limited to 13 published reports since 1980.

**Case Report:**

62-year-old female underwent ESWL targeting 5 mm ureteral stone at proximal left ureter. Following same-day discharge, medication-refractory abdominal pain precipitated emergency room visit where hypotensive with concern for acute abdomen. Of note, patient denied recent trauma to abdominal region. Imaging revealed acute splenic subcapsular hematoma and hemoperitoneum. Transferred to our institution for higher level of care. Hemodynamic instability with ongoing transfusion requirements and diffuse peritonitis dictated emergent procession to operating room, foregoing consideration of splenic artery embolization.

**Conclusions:**

Splenic injury is an exceedingly rare, potentially life-threatening complication of ESWL. We feature the 4th report since 2020 and 14th overall since practice inception in 1980. Despite the resources of a large level 1 trauma center, our case highlights non-operative management may not be pragmatic in the emergent circumstance. Patient and provider education and early recognition remain key to optimize outcomes.

## Case presentation

**Fig. 1:**
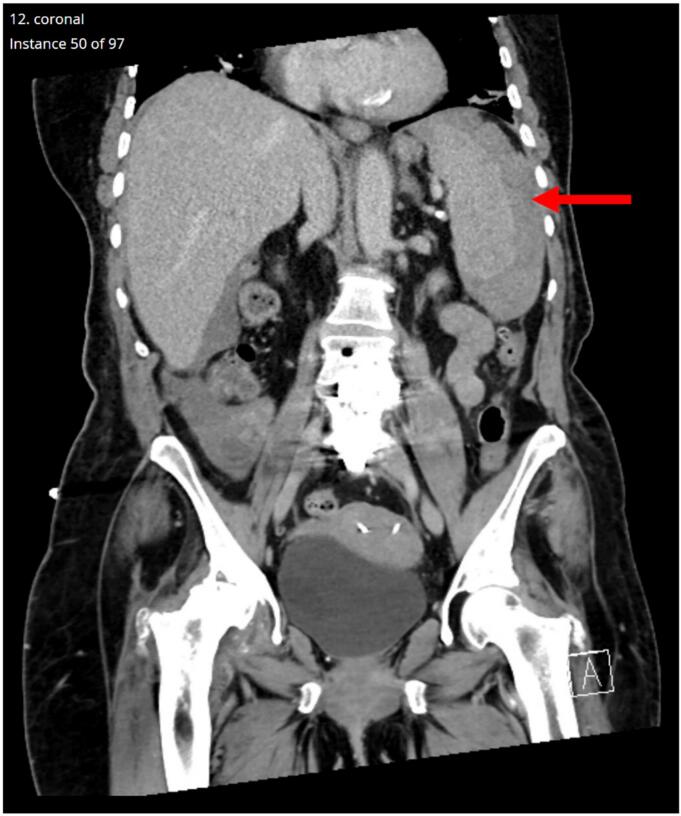
Coronal-view of contrast-assisted CT demonstrating acute splenic subcapsular hematoma (arrow).

On 4/23/25, 62-year-old female with medical history significant for hypothyroidism, gastroesophageal reflux disease, adjustment disorder with mixed anxiety and depressed mood, osteoarthritis, chronic pain, cholelithiasis status post remote cholecystectomy, urinary tract infection (UTI), cystitis, and urolithiasis underwent ESWL targeting 5 mm ureteral stone at proximal left ureter just beyond ureteropelvic junction. Following same-day discharge, she experienced quick resolution of anticipated minimal hematuria though medication-refractory abdominal pain with left shoulder radiation precipitated outside hospital emergency room visit where hypotensive with concern for acute abdomen. Of note, patient denied recent trauma to abdomen or flank region, including falls. Contrast-assisted computed tomography (CT) revealed acute splenic subcapsular hematoma and hemoperitoneum (Fig. 1).

Patient received 1 unit of packed red blood cells ahead of transfer to our institution for higher level of care, interventional radiology and acute care surgery teams engaged enroute. Upon arrival, mildly tachycardic to 110 s with systolic blood pressure in the 80s. Hemoglobin had downtrended to 7.2 from 9.0 over approximately 3-hour span. Physical exam notable for generalized tenderness, including rebound. Hemodynamic instability with ongoing transfusion requirements (2 additional units of packed red blood cells) and diffuse peritonitis dictated emergent procession to operating room, foregoing consideration of splenic artery embolization.

Upon entrance into abdomen, large volume hemoperitoneum encountered. Left upper quadrant was packed preceding Bookwalter retractor placement. Substantial coagulum removed with adherent splenic capsule. Remaining spleen mobilized and medialized facilitating hilum identification and transection with articulating GIA stapler. Evarest patch augmented hilum hemostasis subsequently ensured with copious irrigation. Blake drain placed in left upper quadrant prior to closure. Blood loss estimated at 3000 cc, with intraoperative product resuscitation involving 2 units of packed red blood cells and 1 unit of fresh frozen plasma.

Patient's post-surgical course was unremarkable, with transient low vasopressor requirements limited to the post-anesthesia care unit and a single transfusion of packed red blood cells post-op day 3 secondary to hemoglobin of 7.0. With a stabilized blood panel and blake drain removal following satisfactory output contraction (approximately 100 cc/24 h – serosanguinous in character) among characteristic milestones, discharged on post-op 5 without further concerns at time of paper drafting.

## Discussion

Since advent in 1980, ESWL has become a widely used, noninvasive treatment modality for urolithiasis, impacting up to 11 % of adults in the United States [1]. The complication profile is predominantly limited to manifestations amenable to conservative management, e.g. transient hematuria and ureteric colic. Major hemorrhage is exceedingly rare, especially when splenic in source, with only 13 published reports demonstrating clinically-significant splenic injury [[2], [3], [4], [5], [6], [7], [8], [9], [10], [11], [12], [13], [14], [15]].

While largely speculative given rarity, risk factors for ESWL-induced splenic injury include abnormal spleen growth or position, hypertension (atherosclerosis weakens vascular wall tensile strength raising shock wave vulnerability) [16], coagulopathy (particularly in setting of portal hypertension) [2], and stone locality in left upper calyces. Spleen pathology consequential to cancer, e.g. leukemia and lymphoma, may impose elevated risk [3]. Perhaps the most critical consideration, minimizing movement for precise shock wave administration underscores the importance of noise cancelling headphones [4] and sedation optimization. High BMI (> 30 kg/m^2^) further poses danger if multiple ESWL sessions required for successful fragmentation along with difficult patient positioning and shock wave targeting.

Apart from borderline BMI of 29.3 kg/m^2^, our patient did not have the above risks: no splenomegaly on pre-lithotripsy CT, no precarious stone location, no hypertension, and no coagulopathy (she denies anticoagulant medication). While diagnosed with anxiety, she felt this was appropriately managed via sedative measures and did not endorse any appreciable anxiety-related movement during ESWL.

Treatment for blunt splenic trauma after ESWL should adhere to established guidelines, with non-operative management as the gold standard when feasible [17]. Of the 13 available reports, only one has been managed non-operatively [5]. This patient was hemodynamically responsive to fluid and product resuscitation (2 units each of packed red blood cells, platelets, and fresh frozen plasma) allowing interval splenic artery embolization approximately 12 h after presentation. Dissimilarly, our patient's clinical status and ongoing transfusion requirements precluded further imaging and on-call interventional radiology mobilization. The potential for non-operative management is multifactorial, namely the degree of splenic injury, hemodynamic stability and responsiveness, and interventional radiology timely availability.

Strides continue in ESWL protocol refinement and technological advancement [18]. While low, the risk for inadvertent tissue damage remains, including devastating extrarenal hemorrhage as illustrated. Post-procedural monitoring, particularly when aforementioned risk factors, and education concerning alarm signs, e.g. recalcitrant abdominal pain and sequelae of hypotension, are crucial to prevent delayed care and improve chances of successful non-operative management. Emergency physician awareness must not be overlooked as such cases represent a unique diagnostic challenge with perceivable masquerade as septic shock given triad of hypotension, abdominal pain, and history of UTI.

## Conclusions

Splenic injury is an exceedingly rare, potentially life-threatening complication of ESWL. We feature the 4th report since 2020 and 14th overall since practice inception in 1980. Despite the resources of a large level 1 trauma center, our case highlights non-operative management may not be pragmatic in the emergent circumstance. Patient and provider education and early recognition remain key to optimize outcomes.

## CRediT authorship contribution statement

**Matthew B. Goss:** Project administration, Conceptualization, Formal analysis, Methodology, Supervision, Writing – review & editing, Data curation, Investigation, Writing – original draft. **Joseph S. Banton:** Writing – review & editing, Conceptualization. **Martha M.O. McGilvray:** Conceptualization, Writing – review & editing. **Stephanie M. Armocida:** Conceptualization, Writing – review & editing. **Arvind Kumar:** Writing – review & editing, Conceptualization. **Douglas J.E. Schuerer:** Conceptualization, Writing – review & editing. **Matthew J. McHale:** Writing – review & editing, Conceptualization.

## Funding

No funding was received to conduct this study.

## Declaration of competing interest

The authors declare that they have no known competing financial interests or personal relationships that could have appeared to influence the work reported in this paper.
